# Development and Evaluation of Raloxifene-Hydrochloride-Loaded Supersaturatable SMEDDS Containing an Acidifier

**DOI:** 10.3390/pharmaceutics10030078

**Published:** 2018-06-29

**Authors:** Jong-Hwa Lee, Hak Hyung Kim, Young Ho Cho, Tae-Sung Koo, Gye Won Lee

**Affiliations:** 1 DMPK Group, Korea Institute of Toxicology, Daejeon 305-343, Korea; jhl@kitox.re.kr; 2 Pharvis R&D Department, Pharvis Korea Pharm., Ansan 425-100, Korea; rndscro@gmail.com; 3 Department of Pharmaceutics & Biotechnology, Konyang University, Daejeon 302-832, Korea; micael@konyang.ac.kr; 4 Graduate School of New Drug Discovery and Development, Chungnam National University, Daejeon 305-343, Korea; kootae@cnu.ac.kr

**Keywords:** raloxifene, supersaturatable self-microemulsifying drug delivery system, microemulsion, solubility

## Abstract

Raloxifene hydrochloride (RLH) was formulated into a pH-modified supersaturatable self-microemulsifying drug delivery system (S-SMEDDS) to increase drug solubility and dissolution rate. Optimal formulations of pH-modified S-SMEDDSs were developed by incorporating hydroxypropyl-cellulose-L as a precipitation inhibitor and phosphoric acid as a pH modifier (an acidifier). RLH was dissolved to greater extents by all pH-modified S-SMEDDSs compared with non-pH-modified S-SMEDDSs. In particular, phosphoric acid afforded greater drug dissolution than did the other acidifiers tested, perhaps because phosphoric acid better controlled the pH. More than 50% of the RLH was released from the pH-modified S-SMEDDS at pH 2.5 compared with only ~5% of the drug into aqueous buffer (pH 1.2 or 6.8) after dissolution of a conventional tablet. pH-modified S-SMEDDSs with a hydrophilic polymer and phosphoric acid improved the dissolution behavior of a drug exhibiting poor aqueous solubility.

## 1. Introduction

Raloxifene hydrochloride (RLH, [6-hydroxy-2-(4-hydroxyphenyl)benzo-[b]thien-3-yl][4-[2-(1-piperidiny-ethoxy)-phenyl]ethanone hydrochloride]) is a second-generation selective estrogen receptor modulator [1]. It acts as an estrogen agonist in bone and liver, increasing bone mineral density and reducing the levels of low-density lipoprotein-cholesterol [2,3]. The solubility in water (25 °C) is 0.3 mg/mL. RLH is considered a Biopharmaceutical Classification System (BCS) Class II compound because of its poor aqueous solubility but good permeability [4]. The potential of lipid-based formulations, particularly supersaturatable self-microemulsifying drug delivery systems (S-SMEDDSs), as alternative strategies for delivery of hydrophobic drugs [5] that are poorly water soluble and thus associated with low oral bioavailability has become increasingly recognized [6,7]. SMEDDSs are isotropic mixtures of an oil, a surfactant, a co-surfactant (or solubilizer), and a drug. SMEDDSs become fine oil-in-water microemulsions upon gentle agitation after dilution in water [8]. However, drugs released from microemulsions often precipitate because of decreased solubilities, reducing drug availability and absorption in vivo. Thus, the inhibition of drug precipitation after mixing of SMEDDSs with aqueous solutions and the digestion of lipid components are key considerations when designing such formulations. A supersaturation process can maintain drug solubilization above equilibrium solubility without precipitation [9,10,11,12]. Hydrophilic polymers such as hydroxypropyl methylcellulose (HPMC) and polyvinylpyrrolidone (PVP) can be used in SMEDDS formulations as precipitation inhibitors, forming a supersaturatable self-microemulsifying drug delivery system (S-SMEDDS).

When an S-SMEDDS comes into contact with the aqueous environment of the gastrointestinal tract, the formulation is first emulsified (an emulsion or microemulsion forms immediately), and the drug may be released either in its free form or incorporated into lipid droplets. To enhance drug solubility and to maintain the stability of solid dosage forms by modulating the microenvironment, pH modifiers are added to solid dispersions to greatly enhance drug dissolution by modulating physicochemical factors, including both the microenvironmental pH and intermolecular hydrogen bonding (when an ionizable drug is poorly water soluble) [13,14]. Additionally, to prevent precipitation during dilution with water, a polymeric precipitation agent such as hydroxypropylmethylcellulose (HPMC) may be incorporated into the formulation; this causes the drug to enter a temporary supersaturated state, in turn yielding an S-SMEDDS [15,16].

Here we optimized a pH-modified S-SMEDDS formulation containing RLH, and evaluated the effects of an acidifier and hydrophilic polymer on the inhibition of RLH precipitation. The formulations were evaluated in terms of self-emulsification performance, size, and in vitro drug release and stability.

## 2. Materials and Methods

### 2.1. Materials

RLH was purchased from Sampoong (Seoul, Korea), and Evista^®^ tablets (Eli Lilly and Company, lot A574405) were purchased from a local pharmacy. Propylene glycol monocaprylate (type II) (Capryol™ 90), propylene glycol monocaprylate (type I) NF (Capryol™ PGMC), oleoyl polyoxyl-6 glycerides (Labrafil M^®^ 1944 CS), linoleoyl polyoxyl-6 glycerides (Labrafil M^®^ 2125 CS), caprylocaproyl polyoxyl-8 glycerides (Labrasol^®^), propylene glycol monolaurate (type II) (Lauroglycol™ 90), propylene glycol monolaurate (type I) (Lauroglycol™ FCC), polyglyceryl-3 dioleate (Pluorol^®^ Oleique CC 497), and diethylene glycol monoethyl ether (Transcutol^®^ P) were obtained from Gattefosse (Saint-Priest, France). Polyethylene glycol (PEG)-35 castor oil (Cremophor^®^ EL) and PEG-40 hydrogenated castor oil (Cremophor^®^ RH 40) were obtained from BASF (Hong Kong, China).

PEG sorbitan monolaurate (Tween^®^ 20), PEG sorbitan mono-oleate (Tween^®^ 80), sorbitan monolaurate (Span^®^ 20), sorbitan mono-oleate (Span^®^ 80), PEG, phosphoric acid, citric acid, and tartaric acid were purchased from Daejung (Seoul, Korea). HPMC and hydroxypropylcellulose-L (HPC-L) were purchased from Shin-Etsu (Tokyo, Japan). Acetonitrile and methanol for HPLC were purchased from J.T. Baker (Seoul, Korea).

### 2.2. Solubility Screening Tests

The solubilities of RLH in various oils, surfactants, and co-surfactants were determined. An excess of RLH was added to a glass vial containing 3 mL of vehicle (oil or surfactant) and the mixture stirred using a magnetic stirrer for 2 days at 40 °C. RLH solubilities in mixtures of different surfactant-to-oil ratios were determined by adding excess RLH to such mixtures. Samples were stirred continuously for 2 h at 40 °C and centrifuged (13,000 rpm, 5 min); the supernatants were then filtered through membrane filters (0.45 μm in pore diameter, 13 mm in diameter, Whatman, Pittsburgh, PA, USA). RLH concentrations were determined via HPLC (Shimadzu, Kyoto, Japan); the system featured an LC10-AD isocratic pump, an SPD-10A VP variable spectrophotometric detector, and a Zorbax Eclipse CN column (5 μm pore diameter; 4.6 × 150 mm, Agilent, Carpinteria, CA, USA). The mobile phase consisted of acetonitrile 0.4% *v*/*v* trimethylamine aqueous solution mixture (50:50, *v*/*v*, pH adjusted to 4.0 with phosphoric acid), the flow rate was 2.0 mL/min, and the detection wavelength was 290 nm. The RLH calibration curve was linear (*r* = 0.998) over the concentration range 0.4–50 µg/mL, with the correlation equation being *y* (area) = 7828.4*x* (µg/mL) − 1159.7 (*r* = 0.998). The limits of quantification and detection were 3.26 and 1.08 µg/mL.

### 2.3. Preparation of RLH-Loaded S-SMEDDSs Containing Hydrophilic Polymers

To develop SMEDDSs with high solubilizing capacities, the solubilities afforded by isotropic mixtures of an oil, a surfactant, and a co-surfactant were evaluated. Pseudo-ternary phase diagrams were constructed using Sigmaplot^TM^ (SYSTAT, San Jose, CA, USA) to define the concentration ranges of all components in the microemulsion regions. Mixtures of an oil, a surfactant, and a co-surfactant were prepared at weight ratios of oil to surfactant/co-surfactant mix of 10:90, 20:80, 30:70, 40:60, 50:50, 60:40, 70:30, 80:20, and 90:10. Water was added to each mixture in a dropwise manner, with stirring at 37 °C, until the mixture became clear. RLH-loaded SMEDDS formulations were prepared by addition of RLH (60 mg) to isotropic mixtures of a surfactant (Tween^®^ 20 or Labrasol^®^), an oil (TEC or Capryol™ 90), and a co-surfactant (PEG 200) based on the pseudo-ternary diagrams, mixed by gentle stirring, and heated at 90 ± 5 °C for 30 min. To prepare S-SMEDDS formulations containing hydrophilic polymers, HPMC or HPC-L was added (to 3 and 5% *w*/*w*, respectively) to each of two transparent SMEDDS solutions (Formulations 4 and 9) and then visually assessed in terms of precipitation or phase separation over 24 h. One gram of each S-SMEDDS (containing 60 mg RLH) was added to distilled water, and droplet size distributions were determined via dynamic light scattering (NPA 250, Microtact Inc., York, PA, USA) at 90° and 25 °C.

Transmission electron microscopy (TEM, Tecnai™ G^2^ F30, Fei Company, Oregon, OR, USA) was used to determine the morphology of the microemulsion. After RLH-loaded S-SMEDDS was diluted 100-fold with distilled water, the sample was stained with 2% phosphotungstic acid aqueous solution (PTA) for 5 min at 25 °C. Then, one drop of stained sample was placed on a copper grid. After drying, it was examined under the TEM.

In addition, to determine the effects of different media on the solubility of RLH, 0.2 g of RLH-loaded S-SMEDDS was added to distilled water, SGF (pH 1.2), and SIF (pH 6.8), and was shaken for 24 h at 37 ± 1 °C using a magnetic stirrer. The samples were quantified according to the HPLC method.

### 2.4. Selection of an Acidifier/pH Modifier, and In Vitro Dissolution and Stability Testing

To investigate the effects of acidifiers and to select an optimal acidifier, the pH values of S-SMEDDSs were changed by addition of phosphoric acid, citric acid, and tartaric acid during formulation, and drug dissolution was evaluated in 900 mL SIF (pH 6.8) using a dissolution tester (DRS-14, Campbell Electronics, Mumbai, India) via paddle agitation at 50 rpm at 37 ± 0.5 °C. To investigate the effect of pH on precipitation, the pH values were set to 2.5 ± 0.5, 3.5 ± 0.5, and 4.5 ± 0.5 by adding phosphoric acid to SIF (pH 6.8). Five-milliliter samples were withdrawn from the dissolution tester at 0, 15, 30, 60, 90, and 120 min and analyzed.

In the in vitro release study, a dissolution tester was filled with 900 mL distilled water, SGF (pH 1.2), or SIF (pH 6.8), followed by agitation with a paddle at 50 rpm at 37 ± 0.5 °C. One gram of S-SMEDDSs (Formulations A and B) and pH-modified S-SMEDDSs (Formulations A-1 and B-1) with 60 mg RLH were used to fill hard gelatin capsules (size 0), and the drug release rates were compared with those of a conventional tablet (Evista^®^; with 60 mg RLH). Five-milliliter samples were taken at various time points (0, 5, 15, 30, 60, 90, 120, 180, 240, 300, and 360 min) for analysis; the removed liquid was replaced with the same amount of fresh liquid. Formulations A-1 and B-1, adjusted to pH 2.5 ± 0.5 with phosphoric acid, were finally used to evaluate stability. The pH-modified S-SMEDDS were used to fill hard gelatin capsules (size 0) that were stored under intermediate (25 °C/60% relative humidity (RH)) and accelerated degenerative conditions (40 °C/75% RH) for 3 months. The appearance, drug content, and dissolution rates were evaluated. 

During selection of the acidifiers/modifiers and evaluation of dissolution and stability, all samples were analyzed using an HPLC method.

## 3. Results and Discussion

### 3.1. Solubility Screening Test

When formulating a self-emulsifying preparation, it is important to avoid drug precipitation in the gut lumen in vivo [5]. Thus, the components used should solubilize the drug, and ensure continuing solubility in the ultimate dispersion. Of the various oils, surfactants, and glycerides, TEC, Capryol™ 90, Tween^®^ 20, Tween 80, Labrasol^®^, and PEG 200 exhibited high capacities to solubilize RLH (Table 1). Although Tween^®^ 80 is among these surfactants, it was not chosen because Tween^®^ 80 is present in Evista^®^ tablets. Solubility increases when hydrogen bonds form between RLH and the hydroxyl groups of PEG 200 and attain very high levels when surfactants such as Tween^®^ 20 and Labrasol^®^ are present.

**Figure 1:**
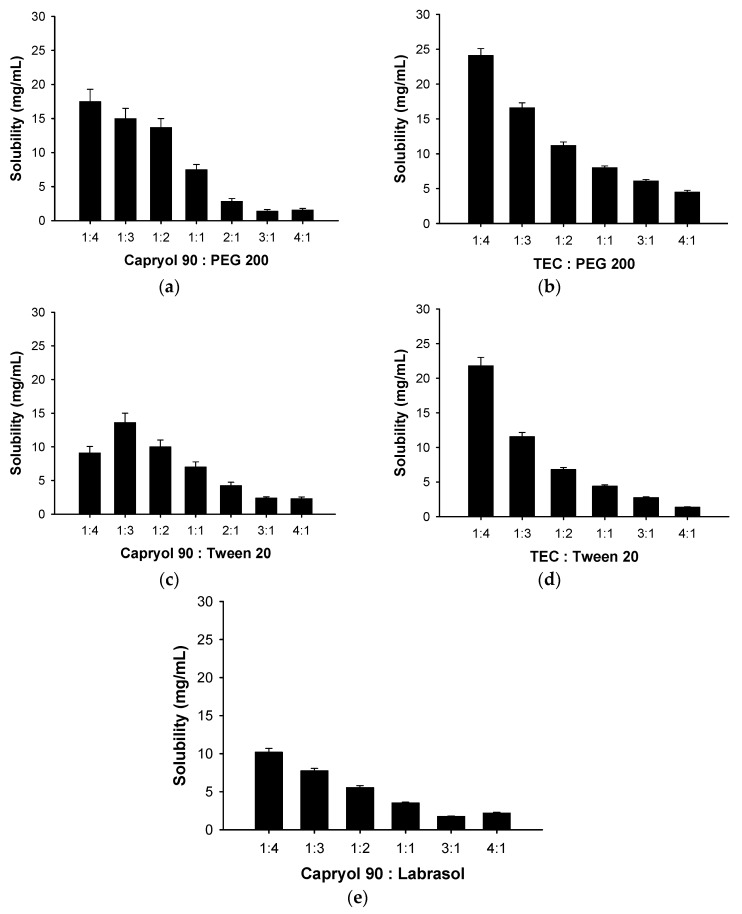
Solubilities of raloxifene hydrochloride in mixtures containing various proportions of (**a**) Capryol™ 90/PEG 200; (**b**) TEC/PEG 200; (**c**) Capryol 90™/Tween^®^ 20; (**d**) TEC/Tween^®^ 20; and (**e**) Capryol™ 90/Labrasol^®^ (means ± SDs, *n* = 3).

RLH solubilities in oils and surfactants and in mixtures of various surfactant/cosurfactant ratios were evaluated (Figure 1, Table 2). The solubilities afforded by mixtures of oils and surfactants (Capryol™ 90/PEG 200, TEC/PEG 200, TEC/Tween^®^ 20, and Capryol™ 90/Labrasol^®^) increased as the ratio of surfactants increased from 1 to 4. In addition, the solubilities afforded by mixtures of Capryol™ 90/Tween^®^ 20 increased as the ratio of surfactant (Tween^®^ 20) increased from 1 to 3. Mixtures of Tween^®^ 20/PEG 200 or Labrasol^®^/PEG 200 afforded high solubilities as the proportion of PEG 200 increased.

To identify SMEDDSs with high solubilizing capacities, the solubilities of RLH afforded by isotropic mixtures of oils, surfactants, and cosurfactants were evaluated (Table 3). Tween^®^ 20 (or Labrasol^®^) and PEG 200 mixtures (Formulations 4 and 13) exhibited remarkable solubilization of RLH (>35 mg/g).

Based on these results, two SMEDDSs using Tween^®^ 20 (or Labrasol^®^) and PEG 200 as S/Cos combinations and TEC (or Capryol™ 90) as the oil were chosen for S-SMEDDS preparation. Formulation A contained TEC, Tween^®^ 20, and PEG 200, and Formulation B contained Capryol™ 90, Labrasol^®^, and PEG 200, as the oil, surfactant, and glyceride, respectively, at a ratio (*w*/*w*) of 5:20:75 based on the result (Table 3).

### 3.2. RLH-Loaded S-SMEDDSs Containing Hydrophilic Polymers

**Figure 2:**
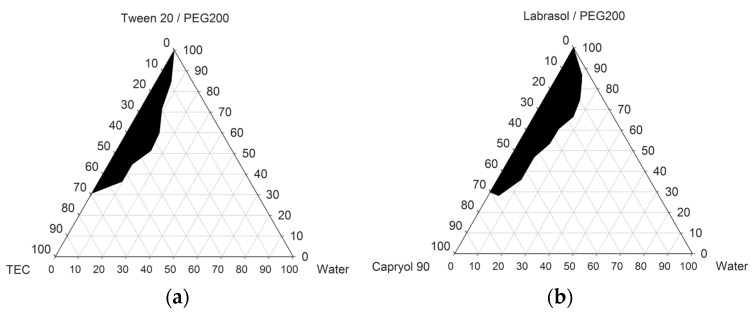
Pseudo-ternary phase diagrams of the microemulsion regions of (**a**) TEC/Tween^®^ 20/PEG 200 and (**b**) Capryol™ 90/Labrasol^®^/PEG 200. Black zones: microemulsion regions.

The oil (TEC or Capryol™ 90) was mixed with S/Cos mixtures of Tween^®^ 20/PEG 200 or Labrasol^®^/PEG 200 at a ratio of 1:3, and the phase behaviors were evaluated (Figure 2). We used the phase diagrams and solubility data to select two SMEDDS formulations with high solubilizing capacities. The type of oil used did not affect the microemulsion regions of the phase diagrams. Generally, PEG 400 can be assumed to act as a co-solvent for poorly soluble drugs, such as fenofibrate, increasing the solubilization capacity of the vehicle. PEG 400, being water-soluble, is anticipated to enter the water phase and redistribute mainly between the water phase and the emulsion–water interface, resulting in a loss of solvent capacity of the vehicle [17]. A similar observation was reported for a composition containing ethanol as a co-solvent [18,19], and finally solved the problem of precipitation by incorporation of a hydrophilic polymer into the system.

S-SMEDDSs (Formulations A and B) made by adding HPC-L and HPMC (to 3 and 5% *w*/*w*, respectively) were assessed. It was found that supersaturatable RLH-loaded SMEDDSs (6% *w*/*v* RLH) could be prepared upon addition of an optimal amount (3% *w*/*v*) of HPC-L; drug re-precipitation did not occur, unlike the case for formulations containing HPMC. The viscosity of dispersion increased as the amount of HPC-L increased from 1 to 5% (*w*/*w*), and dispersion exhibited gel-like properties (1% *w*/*w*, 138 cp; 3%, 495 cp; 5%, 2451 cp) because of the high viscosity, which may hinder self-microemulsification. In S-SMEDDSs containing HPC-L, it is possible that adsorption of the polymer onto crystal surfaces may inhibit crystallization, because the drug may hydrogen bond to the hydrophilic polymer. From this perspective, hydrogen bonds between RLH and HPC-L may slow RLH precipitation after dilution of SMEDDSs [20,21,22,23,24]. In the absence of precipitation during absorption in vivo, the supersaturation phenomenon can maintain drug solubilization above the equilibrium level [9,22]. 

**Figure 3:**
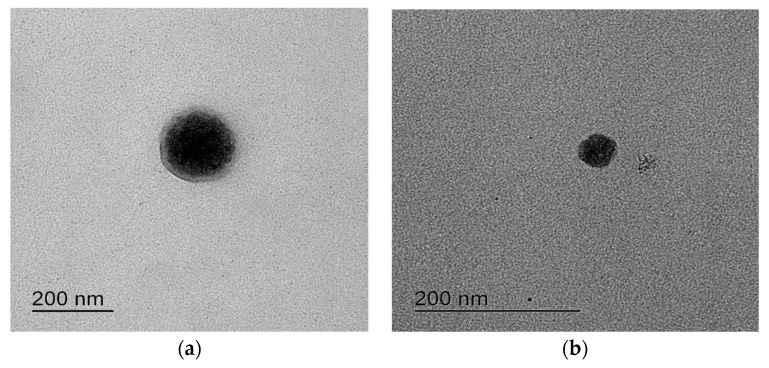
TEM of raloxifene hydrochloride (RLH)-loaded supersaturatable self-microemulsifying drug delivery system (S-SMEDDS) (**a**) Formulation A-1 and (**b**) Formulation B-1.

The S-SMEDDSs dispersed completely and quickly within 20 s (Formulation A, 12.4 s; Formulation B, 17.2 s) upon dilution in water with mild agitation [25]. The average droplet diameters were affected by pH and were less in Formulation B than in Formulation A, as shown in Table 4. The morphological image is shown in Figure 3, in which the droplet was spherical. Saturated solubility of RLH in S-SMEDDS decreased with the increase of pH values (Table 5), indicating that pH affected the solubility like the result in the previous study [26].

### 3.3. Improvement of Dissolution Behavior: Acidifier and Stability Testing

A supersaturatable system is thermodynamically unstable and tends to return to equilibrium via drug precipitation [27]. pH optimization can be used to overcome the stability issues associated with pH-sensitive drugs [28] and to modulate the release of drugs exhibiting pH-dependent solubility [29]. Several authors have successfully enhanced the release (from swellable tablets) of weakly basic drugs using hydrophilic polymers incorporating pH modifiers, such as succinic, fumaric, or adipic acid [30]. The latter materials reduce the microenvironmental pH, enhancing drug solubility and dissolution. 

**Figure 4:**
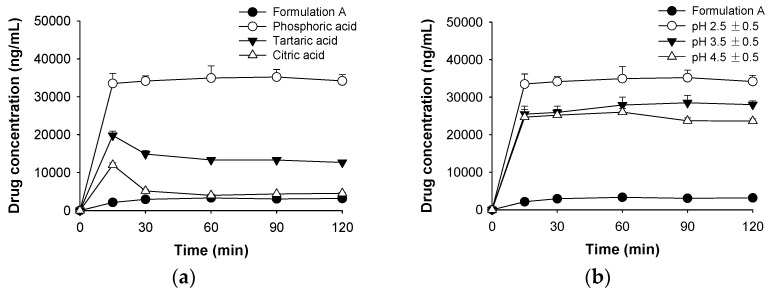
The effects of (**a**) acidifiers (Formulation A (●); phosphoric acid (○), tartaric acid (▼), citric acid (△)) and (**b**) pH modification (Formulation A (●); pH 2.5 (○), pH 3.5 (▼), pH 4.5 (△)) on inhibition of raloxifene hydrochloride precipitation in simulated intestinal fluid (pH 6.8) (means ± SDs, *n* = 3).

The pH of S-SMEDDS (Formulation A) was 6.5 ± 0.5, and RLH was released poorly (less than 5%) into either SGF (pH 1.2) or SIF (pH 6.8). To improve dissolution of RLH, the pH of Formulation A was adjusted to pH 2.5 ± 0.5, 3.5 ± 0.5, and 4.5 ± 0.5 using an acidifier (phosphoric, citric, or tartaric acid). The improved dissolution (into SIF (pH 6.8)) profiles of pH-modified S-SMEDDSs are shown in Figure 4a. RLH was dissolved to greater extents from all pH-modified S-SMEDDSs than from unmodified S-SMEDDSs. In particular, phosphoric acid afforded greater drug dissolution than did the other acidifiers tested, perhaps because phosphoric acid better controlled the pH. The effects of pH 2.5, 3.5, and 4.5 ± 0.5 attained using phosphoric acid on drug precipitation are shown in Figure 4b. As the pH decreased, the amount of dissolved drug released from pH-modified S-SMEDDSs increased, peaking at pH 2.5. This suggests that the drug precipitation/dissolution rate can be improved by modulating the pH of S-SMEDDSs. 

**Figure 5:**
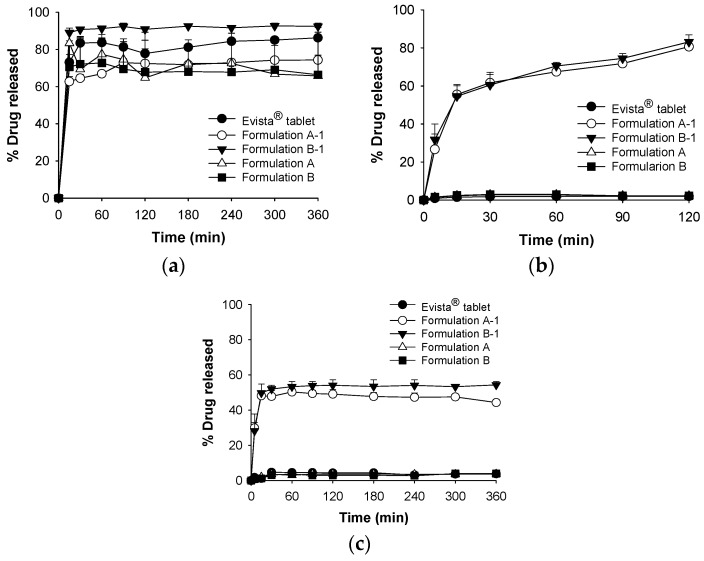
Dissolution profiles of raloxifene hydrochloride contained in Evista^®^ tablets (●) ((Formulations A (△), A-1 (○), B (■), and B-1 (▼)) acidified to (**a**) pH 2.5 using phosphoric acid in distilled water; (**b**) simulated gastric juice (pH 1.2); and (**c**) simulated intestinal fluid (pH 6.8) (means ± SDs, *n* = 3).

We evaluated drug dissolution from S-SMEDDS (Formulations A and B) and pH-modified S-SMEEDS (Formulations A-1, B-1; pH 2.5 ± 0.5) and the profiles of drug release into distilled water, SGF (pH 1.2), and SIF (pH 6.8) (Figure 5). RLH showed poor release (<5%) from tablets and S-SMEDDSs into either SGF (pH 1.2) or SIF (pH 6.8). In contrast, S-SMEDDSs afforded abundant and rapid drug release into water (>60% after 10 min), perhaps because RLH is zwitterionic. Thus, with three pKa values (8.95, 9.83, and 10.91), drug release may be dependent on the pH of the dissolution medium.

The proportions of RLH released from S-SMEDDSs were similar to those released from Evista^®^ tablets into distilled water. RLH was released within 10 min, and the proportions of RLH released from Formulations A and B into distilled water at 6 h decreased to 48.7% and 47.6%, respectively, due to re-precipitation. However, precipitation did not occur when pH-modified S-SMEDDSs were used. The rate of dissolution from pH-modified S-SMEDDSs into SGF (pH 1.2) was high (>80%) compared with that from Evista^®^ tablets (2%).

In stability testing, pH-modified S-SMEDDSs (Formulations A-1 and B-1 with 6% (*w*/*v*) RLH) were stable for 3 months under both intermediate and accelerated storage conditions. Under intermediate conditions (25 °C/60% RH over 3 months), the drug contents (%) were 100.19 and 99.3%, and the time to 80% drug release (T_80%_) into water was <15 min. Under accelerated conditions (40 °C/75% RH for 3 months), the respective figures were 99.64 and 98.21%, and <15 min for the two formulations. The emulsions thus did not change significantly over 3 months. 

The formulations were compatible with capsules made of hard gelatin; no shell deformation, capsular degradation, or compromise in the microemulsifying properties was evident. No phase separation, drug precipitation, or capsule leakage was apparent.

## 4. Conclusions

In summary, pH-modified S-SMEDDS incorporating a precipitation inhibitor (HPC-L) and an acidifier (phosphoric acid) increased the solubility and dissolution rate of RLH. The system at pH 2.5 ± 0.5 readily released the lipid phase to form a fine oil-in-water microemulsion, considerably improving drug release compared with that from the conventional tablet. pH-modified S-SMEDDSs improved the dissolution of RLH, which exhibits poor aqueous solubility.

## Figures and Tables

**Table 1:** The solubilities of raloxifene hydrochloride in various oils and surfactants at 40 °C. All data are means ± SDs (*n* = 3).

Vehicle	Solubility (mg/mL)
Oil	
Triethyl citrate (TEC)	0.58 ± 0.02
Capryol™ 90	0.52 ± 0.02
Glyceryl triacetate	0.38 ± 0.04
Labrafil M^®^ 1944 CS	0.16 ± 0.01
Labrafil M^®^ 2125 CS	0.15 ± 0.00
Lauroglycol™ 90	0.21 ± 0.01
Lauroglycol™ FCC	0.09 ± 0.00
Capryol™ PGMC	0.51 ± 0.02
Pluorol^®^ Oleique CC 497	0.04 ± 0.00
Surfactant	
Cremophor^®^ EL	0.82 ± 0.11
Cremophor^®^ RH 40	1.35 ± 0.48
Labrasol^®^	11.59 ± 0.76
Transcutol^®^ P	8.41 ± 0.62
Span^®^ 20	0.08 ± 0.05
Span^®^ 80	0.01 ± 0.01
Tween^®^ 20	28.38 ± 3.07
Tween^®^ 80	18.78 ± 5.44
Brij^®^ 97	1.44 ± 0.41
Glyceride	
Glycerine	6.50 ± 1.37
Polyethylene glycol (PEG)	9.39 ± 2.21
PEG 200	31.14 ± 1.54
PEG 400	17.86 ± 5.11
Propylene glycol (PG)	11.74 ± 0.55

**Table 2:** The solubilities of raloxifene hydrochloride at various surfactant/cosurfactant ratios (Tween^®^ 20/PEG 200 and Labrasol^®^/PEG 200 ratios) at 40 °C. All data are means ± SDs (*n* = 3).

Ingredients (*w*/*w*, %)			Solubility (mg/g)
Tween^®^ 20	Labrasol^®^	PEG 200	
		100	31.14 ± 1.54
	100		11.59 ± 0.76
100			28.38 ± 3.07
20		80	39.07 ± 0.71
25		75	30.47 ± 2.19
33.3		66.7	30.88 ± 3.39
50		50	27.42 ± 1.19
66.7		33.3	19.68 ± 2.28
75		25	28.28 ± 1.26
80		20	20.68 ± 1.72
	20	80	28.70 ± 0.73
	25	75	24.76 ± 0.76
	33.3	66.7	19.68 ± 2.63
	50	50	11.90 ± 2.07
	66.7	33.3	9.16 ± 0.66
	75	25	10.10 ± 0.75
	80	20	16.12 ± 2.78
25	25	50	26.16 ± 1.94
25	50	25	20.33 ± 2.97

**Table 3:** Solubility of raloxifene hydrochloride in various SMEDDS formulations. All data are means ± SDs (*n* = 3).

No.	Triethyl Citrate	Capryol 90™	Tween^®^ 20	Labrasol^®^	PEG 200	Solubility (mg/g)
1	5		95			23.10 ± 3.07
2	10		90			22.52 ± 0.71
3	5		19		76	32.75 ± 1.76
4	5		20		75	37.96 ± 1.98
5	5		40		55	30.88 ± 0.37
6	5		75		20	22.95 ± 0.04
7	5		30	10	55	31.70 ± 1.87
8	5		27.14	13.57	54.29	15.20 ± 1.44
9	5		23.75	23.75	47.5	23.50 ± 3.19
10	5			20	75	24.48 ± 2.30
11	10			10	80	23.36 ± 0.49
12	10			18	72	29.84 ± 0.84
13		5		20	75	36.83 ± 1.33
14		5		19	76	32.75 ± 1.76
15		10		18	72	24.80 ± 1.82

**Table 4:** Particle size, zeta potential, and polydispersity index of S-SMEDDSs and pH-modified S-SMEDDSs in distilled water. All data are means ± SDs (*n* = 3).

Formulation	Particle Size (nm)	Polydispersity Index	Zeta Potential (mV)
A (S-SMEDDS)	1316.67 ± 72.57	0.18 ± 0.04	33.80 ± 0.75
B (S-SMEDDS)	190.73 ± 2.15	0.25 ± 0.04	54.97 ± 2.14
A-1 (pH-modified S-SMEDDS)	266.57 ± 3.56	0.73 ± 0.01	33.80 ± 1.97
B-1 (pH-modified S-SMEDDS)	195.50 ± 2.90	0.22 ± 0.01	57.23 ± 1.38

**Table 5:** Saturated solubility of RLH from S-SMEDDSs and pH-modified S-SMEDDSs in aqueous media. Values represent the mean ± SD (*n* = 3).

Formulation	Solubility (mg/mL)		
	Water	pH 1.2	pH 6.8
A (S-SMEDDS)	8.89 ± 0.12	0.79 ± 0.19	0.22 ± 0.03
B (S-SMEDDS)	13.13 ± 0.35	2.86 ± 0.42	1.10 ± 0.03
A-1 (pH-modified S-SMEDDS)	11.57 ± 0.45	3.28 ± 0.24	1.95 ± 0.89
B-1 (pH-modified S-SMEDDS)	11.65 ± 0.55	6.05 ± 0.41	3.46 ± 0.15
